# Supplementary material for: Breaking the limitation of mode building time in an optoelectronic oscillator
Source: Nat Commun. 2018 May 9;9:1839. doi: 10.1038/s41467-018-04240-6 (PMC5943580; doi:10.1038/s41467-018-04240-6)
Supplement: Supplementary file 1 — Supplementary Information [file 41467_2018_4240_MOESM1_ESM.pdf]

Supplementary Information for

Breaking the Limitation of Mode Building Time in an Optoelectronic Oscillator

Hao et al.

Breaking the Limitation of Mode Building Time in an Optoelectronic Oscillator

Tengfei Hao[1,2†], Qizhuang Cen[3†], Yitang Dai[3†], Jian Tang[1,2], Wei Li[1,2], Jianping Yao[4*], Ninghua Zhu[1,2*] and Ming Li[1,2*]

†These authors contributed equally to this work.

* *E-mail:* jpyao@uottawa.ca; nhzhu@semi.ac.cn; ml@semi.ac.cn

[1] State Key Laboratory on Integrated Optoelectronics, Institute of Semiconductors, Chinese Academy of Sciences, Beijing 100083, China
[2] School of Electronic, Electrical and Communication Engineering, University of Chinese Academy of Sciences, Beijing 100049, China
[3] State Key Laboratory of Information Photonics and Optical Communications, Beijing University of Posts and Telecommunications, Beijing 100876, China
[4] Microwave Photonics Research Laboratory, University of Ottawa, Ottawa, Ontario K1N 6N5, Canada.

Supplementary Note 1: Theory of the MPF based on PM-IM conversion

Supplementary Figure 1 shows the phase-modulation to intensity-modulation (PM-IM) conversion. The PM-IM conversion is traditionally explained as follows[1, 2]. Assume the phase modulator is driven by a narrow-band radio frequency (RF) signal around Ω, described by $V_{\mathrm{IN}}^{\Omega}(t)e^{-i\Omega t}$. The phase modulation results in three main optical bands, i.e. the carrier and ±1 sidebands. Once one of the sideband is blocked, the photodetector (PD) receives only the carrier and the other sideband, so that the RF signal is recovered. However, there are usually sideband remains after the notch filter, for example, when the notch has limited extinction ratio, or the RF signal or optical source has considerable bandwidth. The mixing product between remains and optical carrier contributes unflatten transfer function of the open-loop cavity, which can be seen as a microwave photonic filter (MPF).

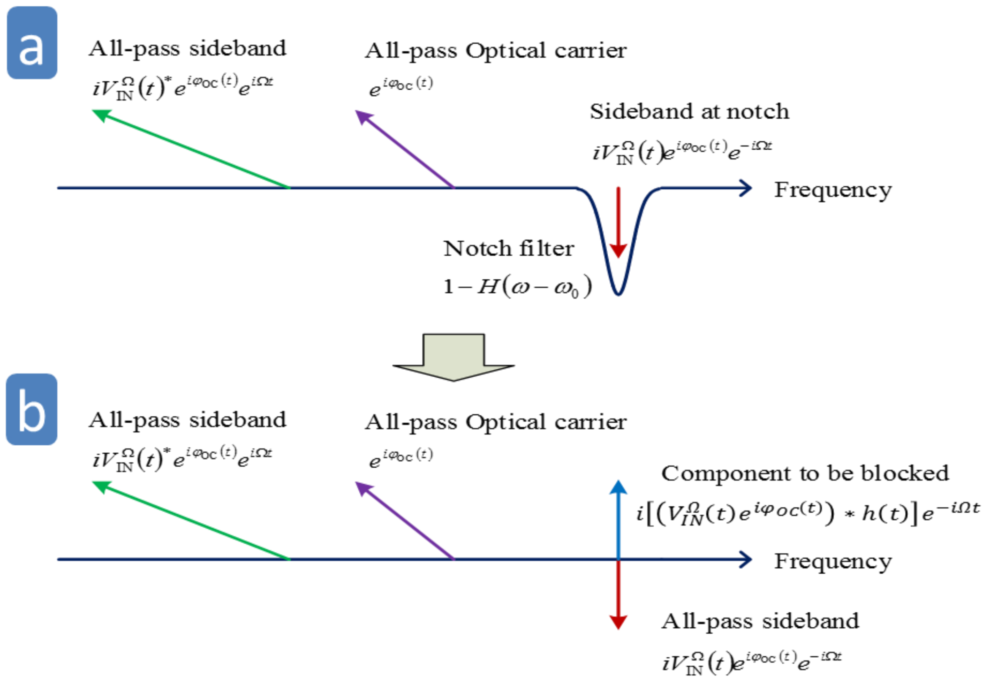

Supplementary Figure 1. Optical spectrum evolution when phase-modulated light wave passes through notch filter. a Traditionally explanation. **b** Equivalent model.

Here we analysis the MPF as follows. Since the notch has usually quite narrow bandwidth while the optical carrier and the other sideband are far away, we assume the notch filter contributes only time delay and loss on them. Detach such delay and loss, transfer function of the notch filter can be described as $1 - H(\omega - \omega_0)$ where ω_0 is the notch center. After notch filter, the optical field, which contains sideband remains, optical carrier, and the other sideband, can also be equivalent to the original three optical bands deconstructed by to-be-blocked component of one sideband, as shown in Fig. S1b. Mathematically, the latter is in proportion to $\left[\left(V_{\mathrm{IN}}^{\Omega}(t)e^{i\varphi_{\mathrm{OC}}(t)}\right) * h(t)\right]$ where $\varphi_{\mathrm{OC}}(t)$ is the phase variation of continuous wave (CW) light source and $h(t)$ is inverse Fourier transform of $H(\omega)$.

At PD the four optical bands are mixed, where the original ±1 sidebands cancel each other exactly due to the phase-modulation-induced opposite sign. The recovered RF signal is then the mixing product of the optical carrier and the to-be-blocked component.

Supplementary Note 2: Oscillation Process of the FDML-OEO

Oscillation process from noise to stable frequency scanning

Supplementary Figure 2 shows the temporal traces of the entire oscillation process from noise to stable frequency scanning measured by a real-time electronic oscilloscope (Tektronics DPO70000, 100 GS/s sampling rate)[3]. The entire process could be divided by initial phase, transition phase and stable frequency scanning phase. Supplementary Figure 2 (a) and (b) shows the frequency distributions and temporal waveforms of the initial and transition phases, respectively. The amplitude of generated temporal waveform is amplified with the increasing of round trips, where the power of real-time frequency distribution is also increased obviously. Finally, in the stable frequency-scanning phase, a linearly chirped microwave waveform with periodical frequency scanning is generated, as shown in Supplementary Fig. 2 (c).

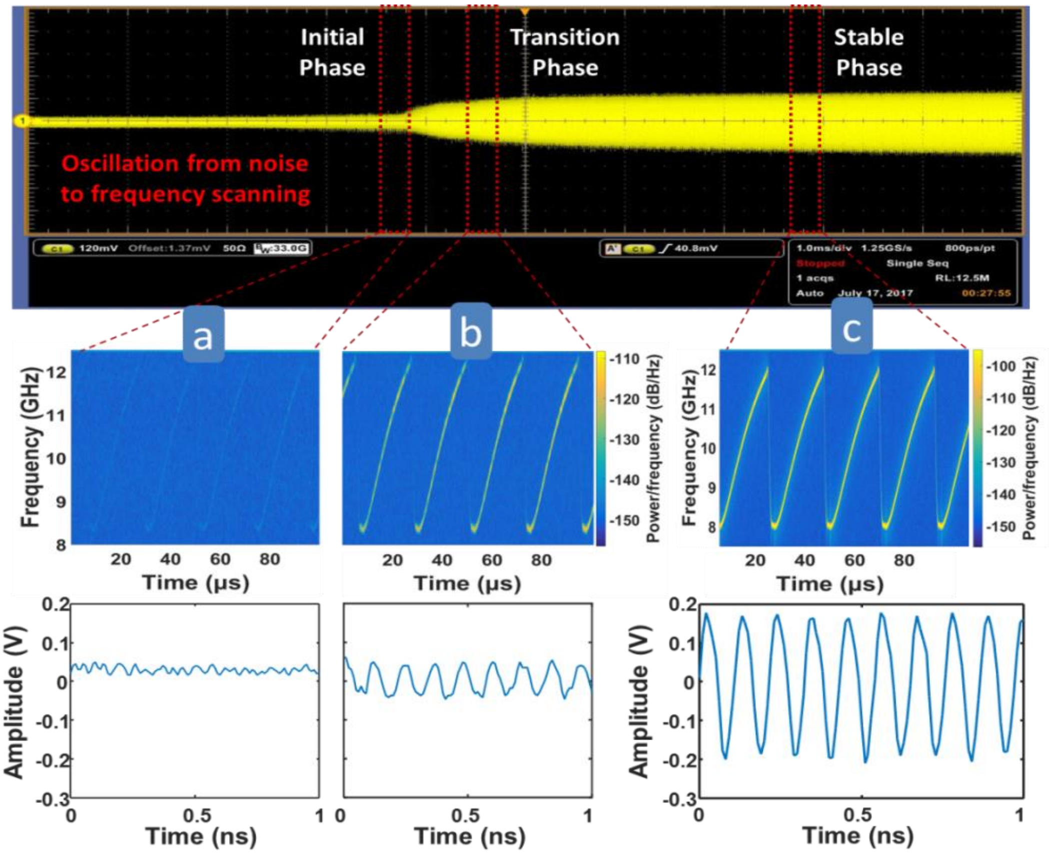

Supplementary Figure 2. Temporal trace of the FDML-OEO oscillation process from noise to stable frequency scanning. a Initial phase. **b** Transition phase. **c** Stable scanning phase.

Oscillation process from single-frequency to stable frequency scanning

The oscillation process from single frequency to stable scanning is also recorded, as

shown in Supplementary Fig. 3. This is achieved by altering injection current of the tunable laser source (TLS) from constant to a saw-tooth driving. Supplementary Figure 3 (a) shows the frequency distributions when the transition process just started. The initial signal frequency is the predominant component during the whole scanning because of injection locking effect. The others scanning components of the waveform is gradually amplified with the increasing of round trips during the transition process, as can be seen in Supplementary Fig. 3 (b), the non-uniform amplification is mainly caused by mode competition. Finally, a stable scanning signal is achieved, as shown in Supplementary Fig. 3 (c).

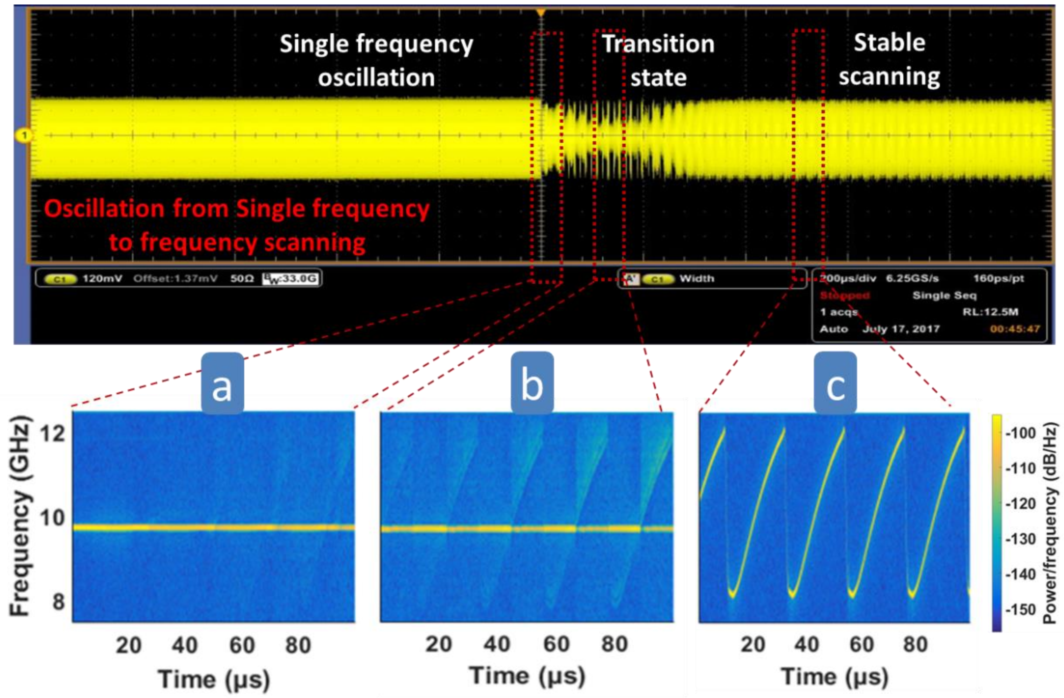

Supplementary Figure 3. Temporal trace of the FDML-OEO oscillation process from signal frequency oscillation to stable frequency scanning. a Transition start. **b** Transition state. **c** Stable scanning.

Supplementary Note 3: Signal Consistency of the of the FDML-OEO

An overlay of 12 traces for frequency scanning microwave waveforms from 8 to 12 GHz recorded in the Fast Frame mode of the Tektronix oscilloscope are shown in Supplementary Fig. 4 (a). The span was set to be 40 μs in order to trace at least one period of the generated waveform. The sampling rate was set to be 25 GS/s rather than 100 GS/s in order to have more frames overlay. The 100,000 times zoom-in view of the overlaid 12 traces is shown in Supplementary Fig. 4 (b). Only very small jitters can be seen from Supplementary Fig. 4, which

indicates a good consistency of the generated chirped microwave waveform.

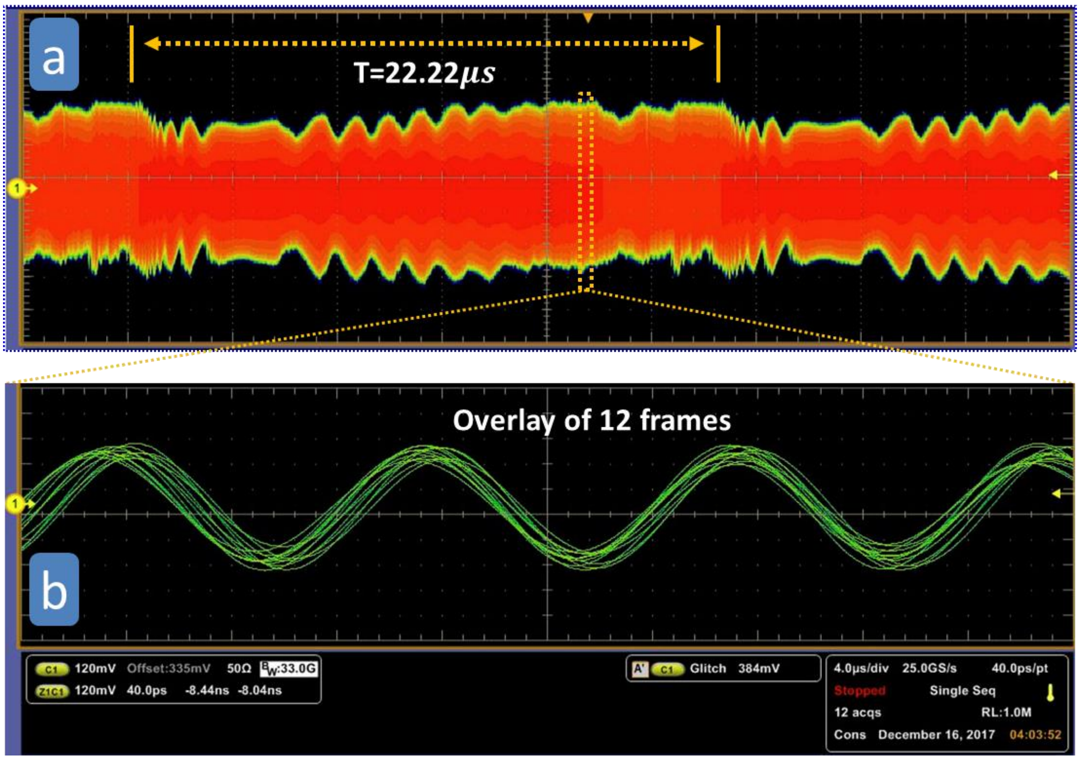

Supplementary Figure 4. Overlay of 12 traces for frequency scanning microwave waveforms from 8 to 12 GHz recorded in the Fast Frame mode of the Tektronix oscilloscope. a In a span of 40 μs. **b** 100,000 times zoom-in view.

Supplementary Note 4: Optical Spectrums of the FDML-OEO

The optical spectrums after the phase-shifted fiber Bragg grating (PS-FBG) notch filter are shown in Supplementary Fig. 5 for the OEO operating at 10 GHz single-mode, 4-6 GHz linearly chirped microwave waveform (LCMW), 8-12 GHz LCMW, and 12-14 GHz LCMW cases, with a resolution of 0.02 nm for all cases. The first-order sideband at the low frequency side was suppressed by the PS-FBG, leaving the first-order sideband at the high frequency side and the optical carrier. The beating between the first-order sideband and the optical carrier generates a microwave signal.

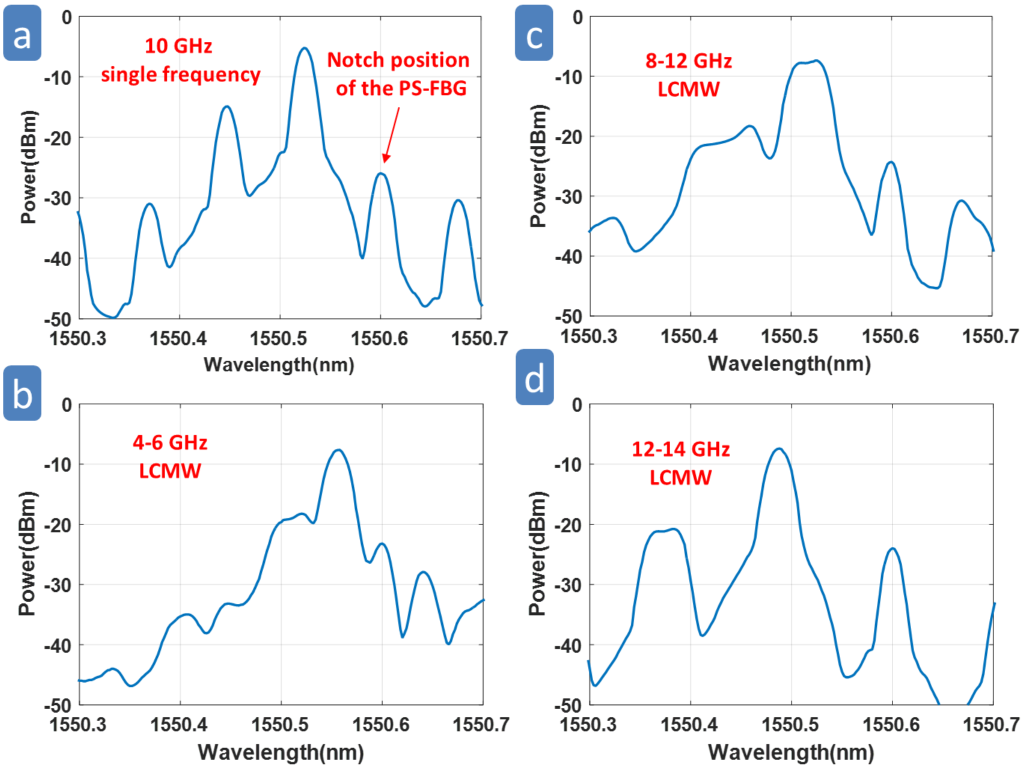

Supplementary Figure 5. Optical spectra from the optical branch after the phase-shifted fiber Bragg grating (PS-FBG) notch filter when the OEO operating at different cases. a 10 GHz single-mode. **b** 4-6 GHz linearly chirped microwave waveform (LCMW). **c** 8-12 GHz LCMW. **d** 2-14 GHz LCMW.

Supplementary Note 5: Phase noise improvement of the FDML-OEO

The relatively large phase noise close to the carrier is mainly caused by the ambient fluctuation because the OEO is sensitive to the environmental changes. Thus, a lower phase noise close to the carrier can be expected by using vibration and thermal isolation.

In addition, the phase locking technique, which is widely used for frequency stabilization of an oscillator, can also be used in our scheme to reduce the phase noise close to the carrier[4]. Basically, an optical self-phase locked loop (SPLL) can be used to stabilize the FDML-OEO. To do so, a portion of the OEO optical output before the PD is coupled out of the OEO loop and delayed by T_D, and the phase of the delayed signal is compared with the phase of the microwave signal generated from OEO. The delay time T_D should satisfy $T_D = lT_{round\text{-}trip}$, where $T_{round\text{-}trip}$ is the round-trip time of the OEO loop and l is an integer. In this way, an error signal is obtained without an external reference oscillator. The error signal is then fed back to the OEO loop to change the effective loop length. The phase noise performance at low offset frequencies as well as the long-term frequency stability can be improved[4].

On the other hand, the phase noise at a frequency-offset far from the carrier is affected by the side-modes of the OEO loop. The side-mode spacing is 45 kHz in our demonstrated system. The side-modes cannot be well suppressed due to the wide bandwidth of the MPF, which is normally at least tens of megahertz. A series of peaks observed from the SSB phase noise measurement, shown in Fig. 7, corresponds to the beating between two adjacent modes which is 45 kHz, and its multiples, leading to a worse phase noise performance at a frequency-offset far from the carrier, as compared with the one from the arbitrary waveform generator (AWG).

A multi-loop OEO is a good candidate[5] to obtain low phase noise at a frequency-offset far from the carrier. Supplementary Figure 6 (a) shows a dual-loop OEO, with both a short loop and a long loop. The modes for the short loop, the long loop, and the dual-loop OEO are shown in Supplementary Fig. 6 (b). The oscillation frequency should satisfy $f_{osc}=k/T_{short\text{-}loop}=m/T_{long\text{-}loop}$, where k and m are both integers and $T_{short\text{-}loop}$ and $T_{long\text{-}loop}$ are round-trip time of the short loop and long loop, respectively. It is can be seen that the side-mode spacing of the OEO is increased by k and m times for a short-loop and long-loop OEO, respectively. So, it is expected that the phase noise performance at a frequency-offset far from the carrier can be improved. In addition, in order to enable a dual-loop OEO to operate with Fourier domain mode locking, the round-trip time of the swept MPF T_{filter} should satisfy the condition such that nT_{filter} equals to the greatest common divisor of $T_{short\text{-}loop}$ and $T_{long\text{-}loop}$, where n is an integer. The sweeping period of the MPF is shortened, as compared with that of a single-loop OEO, leading to an increased chirp-rate for a given scanning bandwidth.

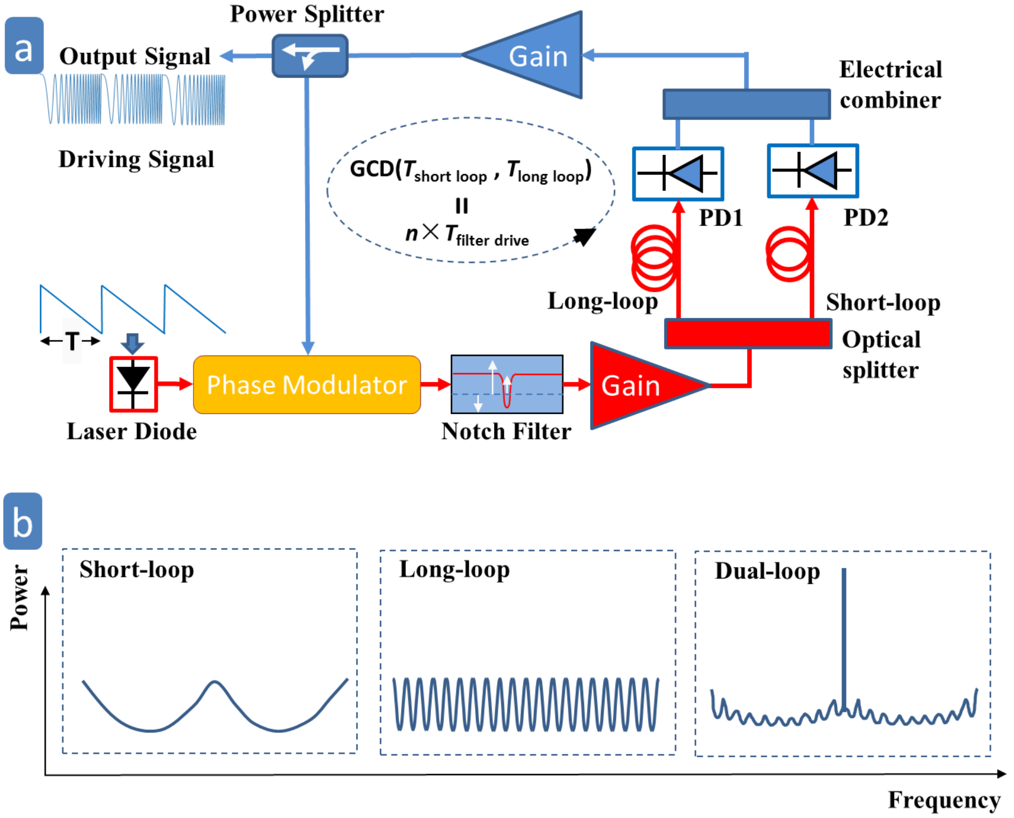

Supplementary Figure 6. a A dual-loop OEO. **b** The oscillation modes for a short-loop, long-loop, and dual-loop OEOs. GCD: greatest common divisor.

Supplementary References

1. Li, W., Li, M., & Yao, J. P. A Narrow-Passband and Frequency-Tunable Microwave Photonic Filter Based on Phase-Modulation to Intensity-Modulation Conversion Using a Phase-Shifted Fiber Bragg Grating. IEEE Trans. Microw. Theory Tech. 60, 1287-1296 (2012).

2. Li, W. & Yao, J. P. A wideband frequency tunable optoelectronic oscillator incorporating a tunable microwave photonic filter based on phase-modulation to intensity-modulation conversion using a phase-shifted fiber Bragg grating. IEEE Trans. Microw. Theory Tech. 60, 1735–1742 (2012).

3. Tektronix MSO/DPO70000 Series Datasheet, https://www.tek.com/datasheet/digital-and-mixed-signal-oscilloscopes.

4. Zhang, L., Poddar, A. K., Rohde, U. L. & Daryoush, A. S. Comparison of optical self-phase locked loop techniques for frequency stabilization of oscillators. IEEE Photon. J. 6, 7903015 (2014).

5. Yao, X. S. & Maleki, L. Multiloop optoelectronic oscillator. IEEE J. Quantum Electron. 36, 79-84 (2000).
